# Supplementary material for: Changes in the Adaptive Cellular Repertoire after Infection with Different SARS-CoV-2 VOCs in a Cohort of Vaccinated Healthcare Workers
Source: Vaccines (Basel). 2024 Feb 23;12(3):230. doi: 10.3390/vaccines12030230 (PMC10974488; doi:10.3390/vaccines12030230)
Supplement: Supplementary file 1 [file vaccines-12-00230-s001.zip › vaccines-2800157-supplementary.pdf]

vaccines

Article

Changes in the Adaptive Cellular Repertoire after Infection with Different SARS-CoV-2 VOCs in a Cohort of Vaccinated Healthcare Workers

Sara Caldrer [1,*], Silvia Accordini [1], Cristina Mazzi [2], Natalia Tiberti [1], Michela Deiana [1], Andrea Matucci [1], Eleonora Rizzi [1], Stefano Tais [1], Fabio Filippo [3], Matteo Verzè [4], Paolo Cattaneo [1], Gian Paolo Chiecchi [1], Concetta Castilletti [1], Massimo Delledonne [5], Federico Gobbi [1] and Chiara Piubelli [1]

[1] Department of Infectious—Tropical Diseases and Microbiology, IRCCS Sacro Cuore—Don Calabria Hospital, Negrar di Valpolicella, 37024 Verona, Italy; silvia.accordini@sacrocuore.it (S.A.); natalia.tiberti@sacrocuore.it (N.T.); michela.deiana@sacrocuore.it (M.D.); andrea.matucci@sacrocuore.it (A.M.); eleonora.rizzi@sacrocuore.it (E.R.); stefano.tais@sacrocuore.it (S.T.); paolo.cattaneo@sacrocuore.it (P.C.); gianpaolo.chiecchi@sacrocuore.it (G.P.C.); concetta.castilletti@sacrocuore.it (C.C.); federico.gobbi@sacrocuore.it (F.G.); chiara.piubelli@sacrocuore.it (C.P.)

[2] Centre for Clinical Research, IRCCS Sacro Cuore—Don Calabria Hospital, Negrar di Valpolicella, 37024 Verona, Italy; cristina.mazzi@sacrocuore.it

[3] Nurse Direction, IRCCS Sacro Cuore Don—Calabria Hospital, Negrar di Valpolicella, 37024 Verona, Italy; fabio.filippo@sacrocuore.it

[4] Medical Direction, IRCCS Sacro Cuore Don—Calabria Hospital, Negrar di Valpolicella, 37024 Verona, Italy; matteo.verze@sacrocuore.it

[5] Department of Biotechnology, University of Verona, 37134 Verona, Italy; massimo.delledonne@univr.it

* Correspondence: sara.caldrer@sacrocuore.it

Supplementary Data

Table S1. Multivariable linear regression models results for the association of VOCs type with B cell subtype.

Outcome	Parameter	Coefficients	95% Confidence Interval (CI)	Outcome	Parameter	Coefficients	95% Confidence Interval (CI)
**Total B cells [CD19+]**	Intercept	3.433	[-5.172; 12.038]	**Switched [CD27+ IgD- IgM-]***	Intercept	37.827	[19.697; 55.957]
	Number of days post vaccination	0.027	[-0.004; 0.057]		**Number of days post vaccination**	0.070	[0.006; 0.134]
	Age	0.075	[-0.059; 0.208]		Age	0.058	[-0.224; 0.34]
	Variant (Ref. VOCD): VOCo	-1.321	[-5.429; 2.787]		Variant (Ref. VOCD): VOCo	-6.410	[-15.065; 2.245]
	Variant (Ref. VOCD): VOCP	-4.087	[-9.168; 0.995]		Variant (Ref. VOCD): VOCP	-5.494	[-16.199; 5.212]
**CD27-**	Intercept	50.215	[26.227; 74.204]	**CD21low CD38low**	Intercept	4.666	[-2.277; 11.608]
	Number of days post vaccination	0.029	[-0.056; 0.113]		Number of days post vaccination	-0.005	[-0.03; 0.019]
	Age	0.296	[-0.077; 0.669]		Age	0.047	[-0.061; 0.155]
	Variant (Ref. VOCD): VOCo	-4.588	[-16.04; 6.864]		Variant (Ref. VOCD): VOCo	2.278	[-1.037; 5.592]
	Variant (Ref. VOCD): VOCP	-5.263	[-19.428; 8.903]		Variant (Ref. VOCD): VOCP	-0.837	[-4.937; 3.262]
**CD27+ memory B cells**	Intercept	49.793	[25.799; 73.786]	**Plasmablasts [Switched B cells CD27 ^{high} CD38high]**	Intercept	-1.312	[-16.166; 13.542]
	Number of days post vaccination	-0.027	[-0.112; 0.058]		**Number of days post vaccination**	0.065	[0.013; 0.118]
	Age	-0.312	[-0.685; 0.061]		Age	0.103	[-0.128; 0.334]
	Variant (Ref. VOCD): VOCo	2.954	[-8.5; 14.408]		Variant (Ref. VOCD): VOCo	-3.290	[-10.381; 3.801]
	Variant (Ref. VOCD): VOCP	5.539	[-8.63; 19.707]		Variant (Ref. VOCD): VOCP	-0.762	[-9.533; 8.01]
**Unswitched [CD27+ IgD- IgM-]**	Intercept	55.062	[35.656; 74.467]	**Transitional B cells [CD27- CD38 ^{high} CD24high]***	Intercept	25.947	[7.934; 43.961]
	Number of days post vaccination	-0.054	[-0.123; 0.014]		Number of days post vaccination	-0.049	[-0.113; 0.015]
	Age	0.019	[-0.283; 0.321]		Age	-0.099	[-0.379; 0.181]

	Variant (Ref. VOCD): VOCo	3.658	[-5.606; 12.922]		Variant (Ref. VOCD): VOCo	7.970	[0.629; 16.569]
	Variant (Ref. VOCD): VOCP	5.301	[-6.158; 16.76]		Variant (Ref. VOCD): VOCP	-0.362	[-10.999; 10.275]

Notes: The models show the correlation of VOCs type with B-cell subtype, adjusted for age and number of days since last vaccination. Linear regression coefficients express the correlation between the parameters and outcome variables, and the sign of the coefficient expresses the direction (positive or negative) of the correlation. Confidence intervals not including zero are to be considered statistically significant. Significant variables are shown in bold.

Table S2. Multivariable linear regression models results for the association of VOCs type with T cell subtype.

Cells	Parameter	Coeff.	95% Confidence Inrterval (CI)	Cells	Parameter	Coeff.	95% Confidence Inrterval (CI)	Cells	Parameter	Coeff.	95% Confidence Inrterval (CI)
CD3	Intercept	13,07	[-6.251; 32.391]	EM-CD4 CD57- PD1-	Intercept	64,99	[36.743; 93.226]	CD8 CD57+ PD1+	Intercept	2,52	[-8.255; 13.295]
	Number of days post vaccination	0,02	[-0.051; 0.085]		**Number of days post vaccination**	-0,11	[-0.212; -0.012]		**Number of days post vaccination**	0,04	[0.001; 0.077]
	Age	0,22	[-0.086; 0.515]		Age	-0,27	[-0.704; 0.174]		Age	0,03	[-0.134; 0.201]
	Variant (Ref. VOCD): VOCo	-3,92	[-13.144; 5.303]		**Variant (Ref. VOCD): VOCo**	29,08	[15.601; 42.565]		Variant (Ref. VOCD): VOCo	-2,58	[-7.722; 2.566]
	**Variant (Ref. VOCD): VOCP**	-11,76	[-23.17; -0.351]		Variant (Ref. VOCD): VOCP	12,78	[-3.896; 29.457]		**Variant (Ref. VOCD): VOCP**	7,41	[1.049; 13.775]
N-CD4	Intercept	18,54	[3.223; 33.854]	EM-CD4 CD57- PD1+	Intercept	18,93	[-5.952; 43.813]	EM-CD8 CD57- PD1-	Intercept	64,02	[32.809; 95.224]
	Number of days post vaccination	0,03	[-0.02; 0.088]		**Number of days post vaccination**	0,11	[0.02; 0.196]		Number of days post vaccination	-0,05	[-0.162; 0.058]
	Age	-0,15	[-0.384; 0.092]		Age	0,25	[-0.135; 0.639]		Age	-0,43	[-0.917; 0.053]
	**Variant (Ref. VOCD): VOCo**	8,26	[0.946; 15.569]		**Variant (Ref. VOCD): VOCo**	-33,33	[-45.21; -21.453]		**Variant (Ref. VOCD): VOCo**	15,58	[0.68; 30.476]
	Variant (Ref. VOCD): VOCP	-5,42	[-14.468; 3.619]		**Variant (Ref. VOCD): VOCP**	-18,84	[-33.537; -4.15]		Variant (Ref. VOCD): VOCP	-6,70	[-25.131; 11.726]
CM-CD4	Intercept	37,44	[19.335; 55.543]	EM-CD4 CD57+ PD1-	Intercept	10,07	[-8.59; 28.727]	EM-CD8 CD57+ PD1+	Intercept	-0,85	[-13.775; 12.067]
	Number of days post vaccination	0,03	[-0.037; 0.09]		Number of days post vaccination	-0,01	[-0.074; 0.058]		**Number of days post vaccination**	0,06	[0.014; 0.105]
	**Age**	0,33	[0.049; 0.612]		Age	-0,08	[-0.369; 0.211]		Age	0,11	[-0.094; 0.307]
	**Variant (Ref. VOCD): VOCo**	-13,98	[-22.627; -5.342]		**Variant (Ref. VOCD): VOCo**	11,60	[2.695; 20.509]		**Variant (Ref. VOCD): VOCo**	-7,18	[-13.347; -1.011]
	Variant (Ref. VOCD): VOCP	-2,69	[-13.378; 8.003]		Variant (Ref. VOCD): VOCP	9,27	[-1.752; 20.284]		**Variant (Ref. VOCD): VOCP**	10,06	[2.428; 17.688]

Subset	Variable	Coef.	95% CI	Subset	Variable	Coef.	95% CI	Subset	Variable	Coef.	95% CI
**CD4 CD57-PD1+**	Intercept	16,31	[1.951; 30.669]	**EM-CD4 CD57+ PD1+**	Intercept	6,02	[-8.571; 20.605]	**TEMRA CD8 CD57- PD1-**	Intercept	35,03	[6.257; 63.807]
	Number of days post vaccination	0,05	[0; 0.101]		Number of days post vaccination	0,01	[-0.039; 0.064]		Number of days post vaccination	-0,01	[-0.109; 0.094]
	Age	0,19	[-0.035; 0.411]		Age	0,09	[-0.135; 0.319]		Age	-0,28	[-0.728; 0.167]
	**Variant (Ref. VOCD): VOCo**	-22,84	[-29.697; -15.987]		**Variant (Ref. VOCD): VOCo**	-7,36	[-14.32; -0.392]		**Variant (Ref. VOCD): VOCo**	17,08	[3.348; 30.821]
	Variant (Ref. VOCD): VOCP	-6,21	[-14.685; 2.273]		Variant (Ref. VOCD): VOCP	-3,20	[-11.818; 5.411]		Variant (Ref. VOCD): VOCP	-7,13	[-24.117; 9.866]
**CD4 CD57- PD1-**	Intercept	70,01	[45.301; 94.724]	**N-CD8**	Intercept	29,05	[8.594; 49.512]	**TEMRA CD8 CD57+ PD1+**	Intercept	8,81	[-6.192; 23.816]
	Number of days post vaccination	-0,01	[-0.098; 0.077]		Number of days post vaccination	0,03	[-0.041; 0.103]		Number of days post vaccination	0,05	[0.001; 0.107]
	Age	-0,25	[-0.632; 0.136]		Age	-0,39	[-0.707; -0.071]		Age	-0,04	[-0.269; 0.198]
	**Variant (Ref. VOCD): VOCo**	25,45	[13.648; 37.242]		**Variant (Ref. VOCD): VOCo**	6,98	[-2.784; 16.749]		**Variant (Ref. VOCD): VOCo**	-8,57	[-15.734; -1.408]
	Variant (Ref. VOCD): VOCP	9,46	[-5.135; 24.05]		Variant (Ref. VOCD): VOCP	-4,61	[-16.695; 7.467]		Variant (Ref. VOCD): VOCP	-2,17	[-11.031; 6.689]

Notes: The models show the correlation of VOCs type with T-cell subtype, adjusted for age and number of days since last vaccination. Linear regression coefficients express the correlation between the independent and dependent variables, and the sign of the coefficient expresses the direction (positive or negative) of the correlation. Confidence intervals that not including zero are to be considered statistically significant. Significant variables are shown in bold.

Table S3. Immunophenotype for T cells stratified for SARS-CoV-2 VOCs depending on the timing between vaccination and infection.

**Infection within 90 days from last vax**	**NO** (n = 13)					**YES** (n = 22)				
**% T cells subset**	**VOCP** N = 6[1]	**VOCD** N = 3[1]	**VOCO** N = 4[1]	**p-value**[2]	**Adj. p-value**[3]	**VOCP** N = 3[1]	**VOCD** N = 9[1]	**VOCO** N = 10[1]	**p-value**[2]	**Adj. p-value**[3]
LEUKOCYTES (CD45+)	15 [13- 21]	24 [12- 24]	10 [9- 12]	0.177	0.432	21 [19- 21]	22 [3- 41]	21 [12- 27]	0.929	0.939
CD3+	7 [6- 15]	15 [14- 17]	27 [23- 31]	0.059	0.376	14 [11- 15]	28 [24- 34]	17 (12, 24)	0.098	0,339
CD4+	41 [30- 47]	56 [52- 58]	52 [43- 55]	0.238	0.466	27 [26- 38]	51 [44- 58]	55 (45, 59)	0.149	0,339
CD8+	48 [43- 58]	31 [29- 31]	35 [33- 45]	0.044	0.376	58 [46- 63]	39 [34- 51]	36 (27, 41)	0.163	0,339
TCM-CD8+	8.0 [7.3- 10.0]	12.4 [11.7- 18.7]	9.3 [7.2- 10.7]	0.115	0.431	5 [4- 6]	12 [7- 14]	13 [8- 16]	0,146	0,356
TEMRA-CD8+	30 [19- 44]	16 [15- 27]	8 [5- 11]	0.040	0.376	33 [24- 33]	25 [15- 33]	22 (13, 29)	0,383	0,561
TEM-CD8+	50 [45- 61]	42 [39- 44]	66 [54- 74]	0.343	0.587	58 [55- 61]	44 [41- 54]	45 [38- 58]	0,279	0,477

T_N-CD8+	9 [6- 14]	14 [13- 23]	14 [6- 29]	0.458	0.662	1 [1- 15]	18 [10- 21]	18 (13, 32)	0,286	0,477
CD8+ (CD57-/PD1-)	29 [26- 34]	41 [38- 48]	57 [34- 75]	0.290	0.513	26 [18- 45]	47 [26- 52]	46 (40, 61)	0,5	0,612
CD8+ (CD57-/PD1+)	14 [12- 17]	37 [31- 37]	10 [7- 14]	0.041	0.376	10 [8- 14]	19 [17- 24]	17 (11, 23)	0,317	0,501
CD8+ (CD57+/PD1-)	39 [23- 46]	15 [13- 17]	23 [12- 45]	0.236	0.466	38 [29- 55]	20 [10- 33]	29 (12, 34)	0,533	0,64
CD8+ (CD57+/PD1+)	14 [12- 20]	8 [8- 9]	4 [2- 5]	0.038	0.376	12 [9- 15]	10 [7- 13]	8 (5, 12)	0,455	0,612
T_{EM}-CD8+ (CD57-/PD1-)	32 [25- 45]	48 [41- 52]	49 [40- 56]	0.519	0.662	31 [19- 44]	30 [25- 41]	56 (41, 67)	0,081	0,311
T_{EM}-CD8+ (CD57-/PD1+)	17 [14- 30]	38 [28- 41]	32 [26- 46]	0.407	0.627	17 [14- 24]	27 [16- 36]	23 (13, 25)	0,69	0,748
T_{EM}-CD8+ (CD57+/PD1-)	26 [14- 34]	10 [9- 16]	12 [8- 14]	0.133	0.432	17 [16- 38]	18 [6- 36]	16 (5, 23)	0,569	0,659
T_{EM}-CD8+ (CD57+/PD1+)	19 [17- 23]	6 [5- 9]	4 [3- 5]	0.007	0.376	21 [15- 23]	12 [10- 17]	3 (2, 9)	**0,024**	0,157
T_{EMRA}-CD8+ (CD57-/PD1-)	13 [11- 18]	23 [22- 25]	53 [35- 64]	0.170	0.432	14 [9- 21]	21 [16- 22]	30 (23, 44)	0,115	0,339
T_{EMRA}-CD8+ (CD57-/PD1+)	79 [65- 84]	43 [37- 54]	35 [31- 49]	0.203	0.466	64 [64- 77]	48 [42- 68]	57 (29, 70)	0,382	0,561
T_{EMRA}-CD8+ (CD57+/PD1-)	3 [2- 4]	15 [10- 25]	6 [1- 12]	0.173	0.432	5 [3- 5]	7 [5- 10]	4 (2, 16)	0,409	0,584
T_{EMRA}-CD8+ (CD57+/PD1+)	7 [4- 10]	10 [7- 16]	2 [1- 3]	0.094	0.413	5 [4- 11]	15 [11- 26]	4 (2, 12)	0,087	0,311
T_{CM}-CD4+	55 [49- 57]	60 [51- 63]	44 [41- 46]	0.216	0.466	53 [45- 56]	57 [49- 68]	47 (37, 50)	0,079	0,311
T_{EMRA}-CD4+	0.38 [0.18- 0.78]	0.13 [0.13- 1.03]	0.44 [0.39- 1.09]	0.617	0.726	0.88 [0.76- 0.98]	0.50 [0.16- 0.61]	1.38 (0.35, 2.39)	0,256	0,465
T_{EM}-CD4+	36 [28- 40]	16 [14- 29]	38 [33- 44]	0.390	0.615	45 [41- 47]	17 [15- 26]	28 (16, 36)	0,248	0,465
T_N-CD4+	11.4 [9.6- 13.6]	18.9 [16.5- 22.9]	15.9 [14.6- 19.0]	0.061	0.376	**2 [2- 8]**	**17 [12- 19]**	**25 (18, 35)**	**0,009**	0,099
CD4+ (CD57-/PD1-)	68 [64- 72]	59 [56- 66]	94 [86- 95]	0.122	0.431	**63 [56- 66]**	**60 [44- 65]**	**87 (78, 92)**	**0,009**	0,099
CD4+ (CD57-/PD1+)	26 [18- 28]	25 [24- 30]	3 [1- 5]	0.089	0.413	**23 [22- 28]**	**34 [32- 39]**	**10 (4, 12)**	**0,001**	**0,039**
CD4+ (CD57+/PD1-)	3.1 [1.8- 4.2]	0.9 [0.8- 3.0]	1.3 [0.3- 7.0]	0.519	0.662	5.3 [5.0- 6.1]	1.2 [0.8- 2.1]	1.2 (0.4, 1.6)	0,154	0,356
CD4+ (CD57+/PD1+)	4.5 [2.2- 6.4]	5.1 [3.3- 12.2]	2.1 [1.6- 4.2]	0.741	0.824	9.4 [6.3- 11.3]	3.9 [2.3- 4.5]	2.1 (1.2, 3.1)	0,115	0,339
T_{CM}-CD4+ (CD57-/PD1-)	81 [80- 82]	69 [66- 74]	84 [70- 88]	0.162	0.432	78 [77- 83]	67 [66- 74]	77 (64, 81)	0,071	0,311
T_{CM}-CD4+ (CD57-/PD1+)	1.40 [1.05- 1.91]	0.68 [0.56- 0.72]	0.40 [0.37- 0.65]	0.063	0.376	0.96 [0.70- 1.15]	0.54 [0.33- 0.55]	0.87 (0.45, 1.24)	0,232	0,465
T_{CM}-CD4+ (CD57+/PD1-)	17 [14- 18]	29 [25- 32]	15 [11- 28]	0.171	0.432	20 [15- 21]	31 [25- 32]	21 (18, 31)	**0,051**	0,278
T_{CM}-CD4+ (CD57+/PD1+)	1.54 [1.07- 2.25]	0.62 [0.55- 0.78]	0.56 [0.37- 0.94]	0.177	0.432	1.01 [0.72- 1.15]	0.93 [0.65- 1.09]	0.64 (0.56, 1.26)	0,866	0,896
T_{EM}-CD4+ (CD57-/PD1-)	62 [55- 72]	38 [38- 58]	74 [64- 82]	0.290	0.513	**45 [41- 52]**	**26 [19- 39]**	**62 (54, 77)**	**0,002**	**0,039**
T_{EM}-CD4+ (CD57-/PD1+)	20 [4- 35]	37 [21- 48]	5 [1- 15]	0.252	0.473	**22 [15- 26]**	**52 [51- 58]**	**8 (2, 14)**	**<0.001**	**0,023**

T_{EM}-CD4+ (CD57+/PD1-)	12 [3- 19]	1 [0- 8]	15 [13- 17]	0.241	0.466	**16 [12- 24]**	**1 [1- 5]**	**10 (9, 29)**	**0,01**	0,099
T_{EM}-CD4+ (CD57+/PD1+)	4.3 [3.1- 5.1]	3.4 [2.6- 13.9]	2.0 [1.4- 2.7]	0.180	0.432	18 [11- 20]	11 [4- 21]	4 (2, 5)	0,202	0,417

Notes: [1] Continuous variables are expressed as: median [IQR]. [2] Kruskal-Wallis rank sum test. [3] False discovery rate correction for multiple testing. Statistically significant comparisons are shown in bold.

Supplementary Figures

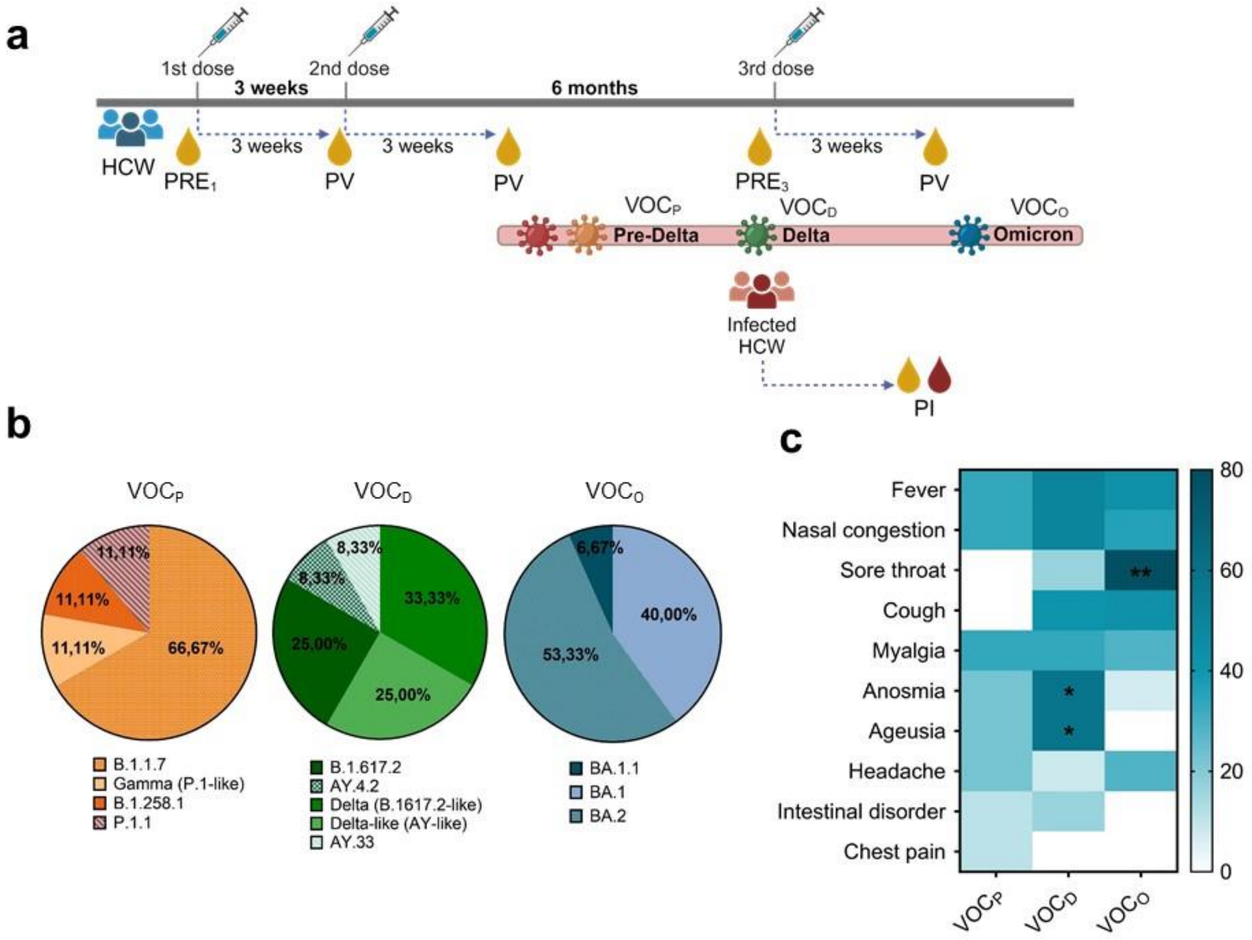

Figure S1. Population characteristic according to VOCs**. (a)** The schematic time-line describes the vaccination schedule-the infection period and the timing for samples harvests. **(b)** Pie charts describing the distribution of the SARS-CoV-2 lineage (expressed as percentage of distribution) across the three groups of HCWs affected by different VOCs- i.e. Pre-Delta (Orange, n=9)- Delta (Green, n=12)- or Omicron (Blue, n=15). **(C)** Relative frequency distribution (%) of symptoms in HCWs according to the VOC.

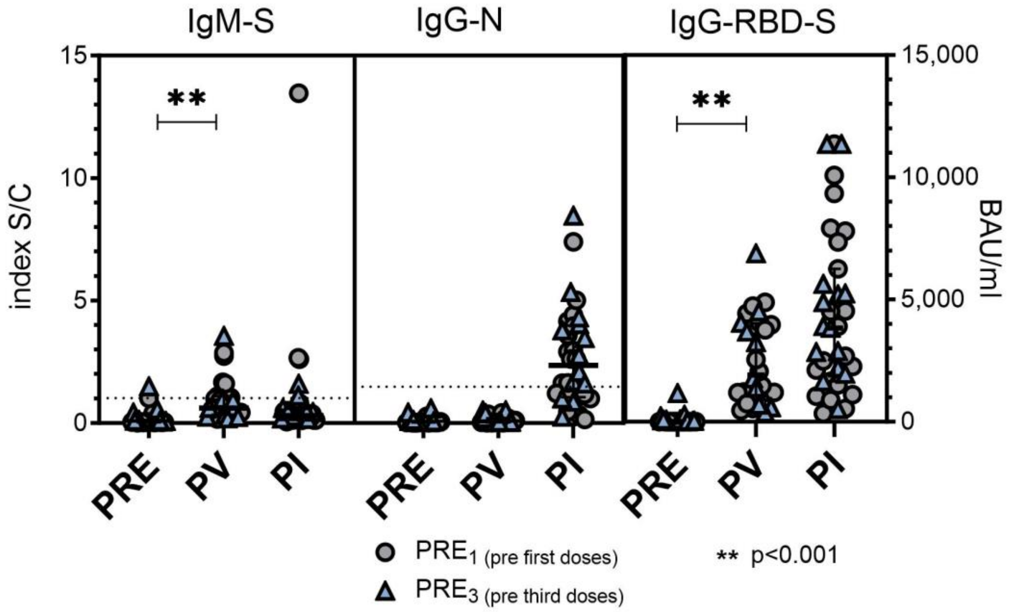

Figure S2. SARS-CoV-2 Antibody levels. Scattered plot representing the different levels of IgM-S, IgG-N(expressed in index S/C)and the SARS-CoV-2 IgG-RBD-S levels (expressed in BAU/ml) at different time points: pre-vaccination (pre first dose-PRE1 or pre third dose- PRE3), post-vaccination (PV) and after SARS-CoV-2 VOCs infection (PI). The bold line on each box indicates the median and IQR. Statistical significance were evaluated by Wilcoxon signed rank test.
